# Peripheral Ossifying Fibroma: A Case Report

**DOI:** 10.7759/cureus.59749

**Published:** 2024-05-06

**Authors:** Soya Alfred Xavier, Kandhal Yazhini P

**Affiliations:** 1 Oral and Maxillofacial Surgery, Saveetha Dental College and Hospitals, Saveetha Institute of Medical and Technical Sciences, Saveetha University, Chennai, IND

**Keywords:** hyperplastic growth, surgical excision, gingival irritation, peripheral ossifying fibroma, inflammatory growth

## Abstract

Peripheral ossifying fibroma (POF) is a reactive overgrowth that most commonly occurs on the gingiva. It is a benign oral soft tissue tumour. It is most commonly found on the anterior maxilla and has a female predilection. Most commonly found to occur in the second decade of life. This type of lesion originates from the cells of the periodontal ligament. It is often associated with trauma or local irritants, such as subgingival plaque and calculus, dental appliances and poor-quality dental restorations. This entity requires a proper treatment protocol and a regular follow-up. It can cause significant discomfort and irritation in the oral health if left untreated. The recurrence rate of the lesion varies according to the authors. This case report describes a case of POF in an adult female patient which was treated using surgical excision resulting in an uneventful healing during the post-operative period.

## Introduction

Peripheral ossifying fibroma (POF) is also known as calcifying or ossifying fibroid epulis, peripheral cemento-ossifying fibroma and peripheral fibroma with calcification. It is a type of localised reactive lesion which occurs on the gingiva. It is a non-neoplastic reactive lesion, which can be soft with a smooth surface. Statistics state that 60% of the lesions occur in the maxillary anterior region [1]. The colour of the lesion can vary from light rosy colour to dark cherry red colour. A common site of occurrence is the inter-dental papilla. The pathogenesis of POF is unclear; however, a proposed explanation for the lesion is that it originates from the periodontal ligament (PDL) due to irritant factors like plaque, calculus, trauma and microorganisms. It occurs most commonly in females in the second to fourth decade of life [2]. In most cases radiographically no changes are observed; however, sometimes areas of radiopacity may be observed. Treatment options include early detection, surgical intervention and thorough oral prophylaxis. The recurrence rate of the lesion ranges from 8% to 20%, the most common cause of recurrence being incomplete surgical excision and the presence of irritants [1]. This case reports an atypical location, posterior mandibular region where POF was present.

## Case presentation

A 50-year-old female patient reported to the Department of Oral and Maxillofacial Surgery complaining of intra-oral swelling on the right side of the posterior mandibular gingiva for the past five months. The patient complained of difficulty in speech and mastication because of the swelling. History revealed that swelling has been small in size but has gradually increased to the present size over a period of time. Bleeding from the site was also observed during the brushing of the teeth. The patient had no medical history.

**Figure 1:**
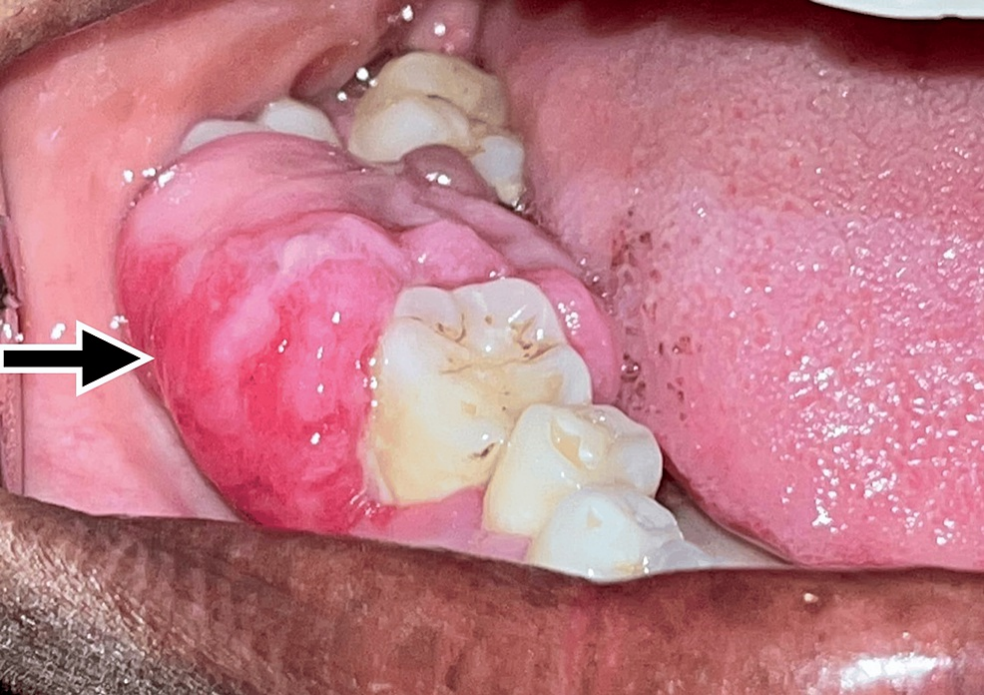
Preoperative intraoral view showing the lesion on the right gingival region

Extra-oral examination revealed no gross asymmetry. Regional lymph nodes were not palpable. Intra-oral examination revealed the presence of firm, mobile, non-tender, soft tissue growth of size 2.1 x 1.5 x 1.2 cm seen extending from the lower right buccal alveolar mucosa to lingual alveolar mucosa on 46 to 48 gingival region. A labial tilted second molar was seen, around which the soft tissue growth was seen (Figure 1). Mobility of the teeth was seen with respect to 46, 47 and 48.

**Figure 2:**
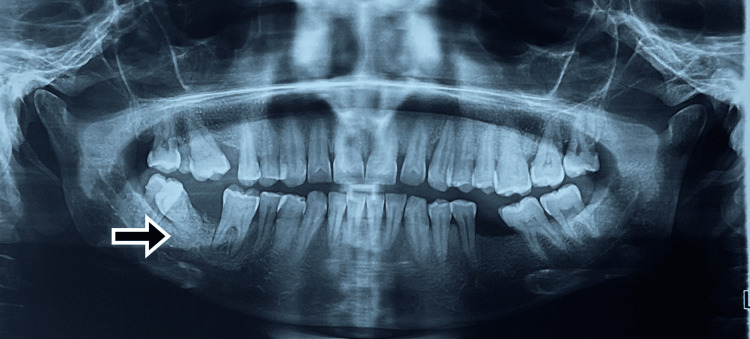
Orthopantomogram showing tilted right second molar

The orthopantomogram revealed soft tissue shadow between 46 and 47 regions. No radiopacities were observed. A labial tilted right mandibular second molar tooth was seen (Figure 2). Based on the history, clinical presentation, and radiological investigation, the lesion was given a differential diagnosis of fibroma, peripheral odontogenic fibroma and periphery ossifying fibroma. A surgical excision was planned.

**Figure 3:**
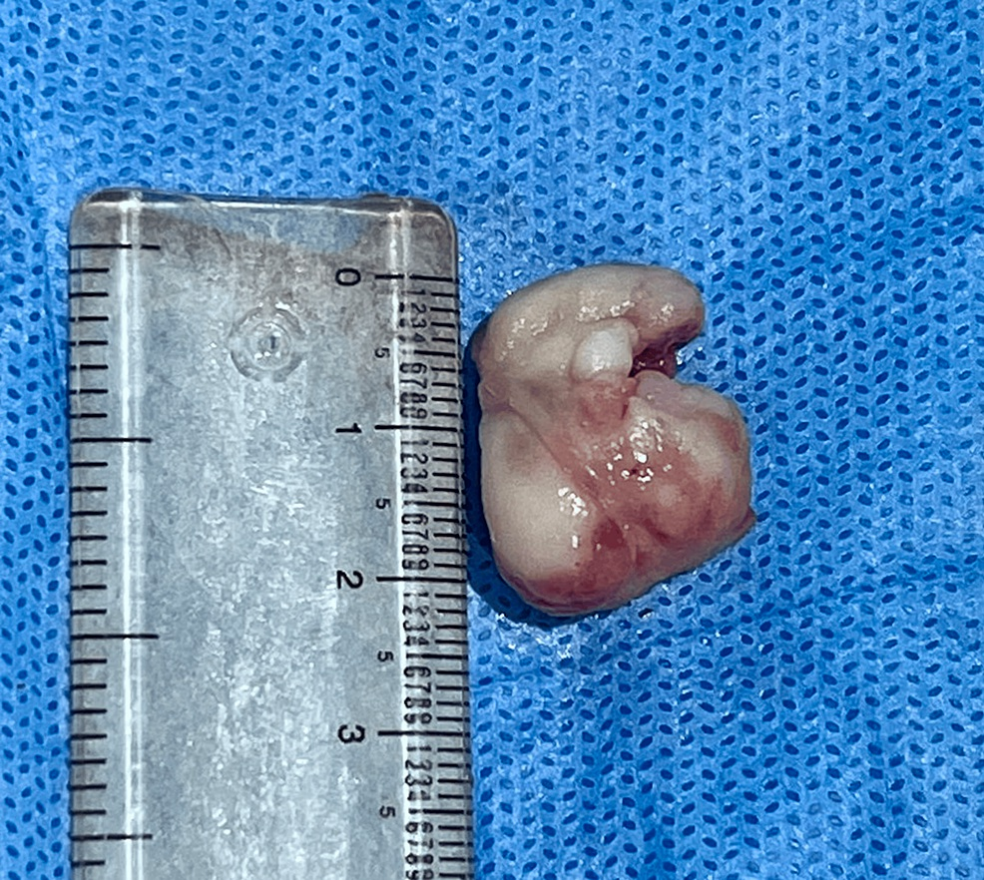
Excised specimen measuring 2.1 x 1.5 cm

Informed consent was obtained from the patient for the procedure. Under local anaesthesia, using a no. 15 surgical blade, incision was given on the base of the lesion and excised (Figure 3). Mucoperiosteal flap elevation was done in relation to 48, 47 and 46 followed by extraction of 46, 47 and 48. Thorough curettage was done in the depth of the lesion and surrounding tissues. Ethicon bone wax was placed on the bone to arrest bleeding. Trusilk 3-0 black braided silk sutures were placed and hemostasis was achieved. The patient was prescribed antibiotics, analgesics and chlorhexidine mouthwash.

**Figure 4:**
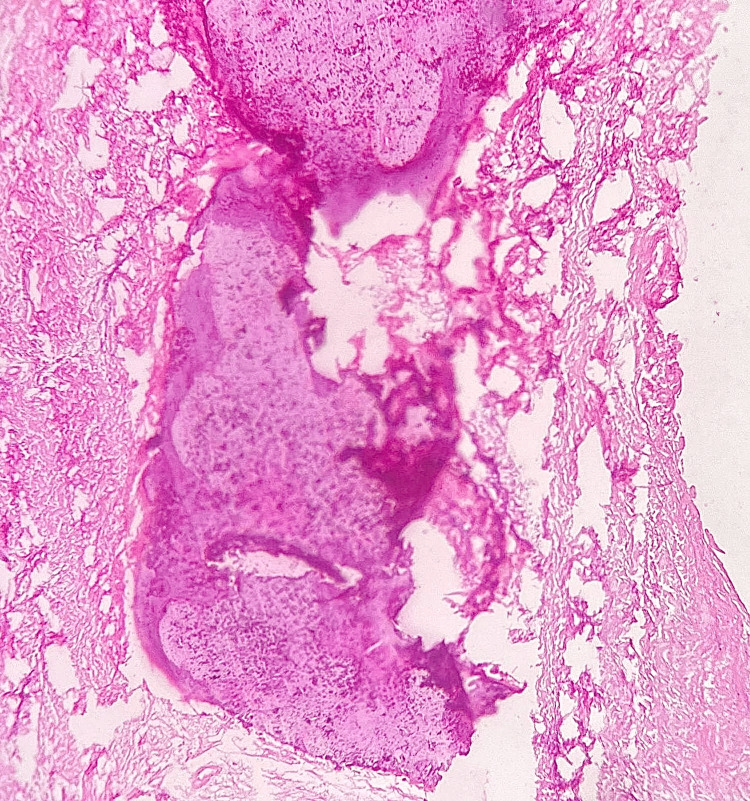
Histopathological findings revealing features suggestive of peripheral ossifying fibroma

The haematoxylin and eosin-stained excised specimen was sent for histopathological examination. The stained sections under 10X microscopic view showed fibrous connective tissue stroma composed of numerous plump fibroblasts along with areas of ossification with osteocytic lacunae containing osteocytes. There was also the presence of a few acellular basophilic calcified areas resembling cementum. The overlying para-keratinised stratified squamous epithelium was of variable thickness indicating pseudoepitheliomatous hyperplasia in a few areas (Figure 4). Correlating clinically, radiographically and histopathologically, the diagnosis was confirmed as POF.

**Figure 5:**
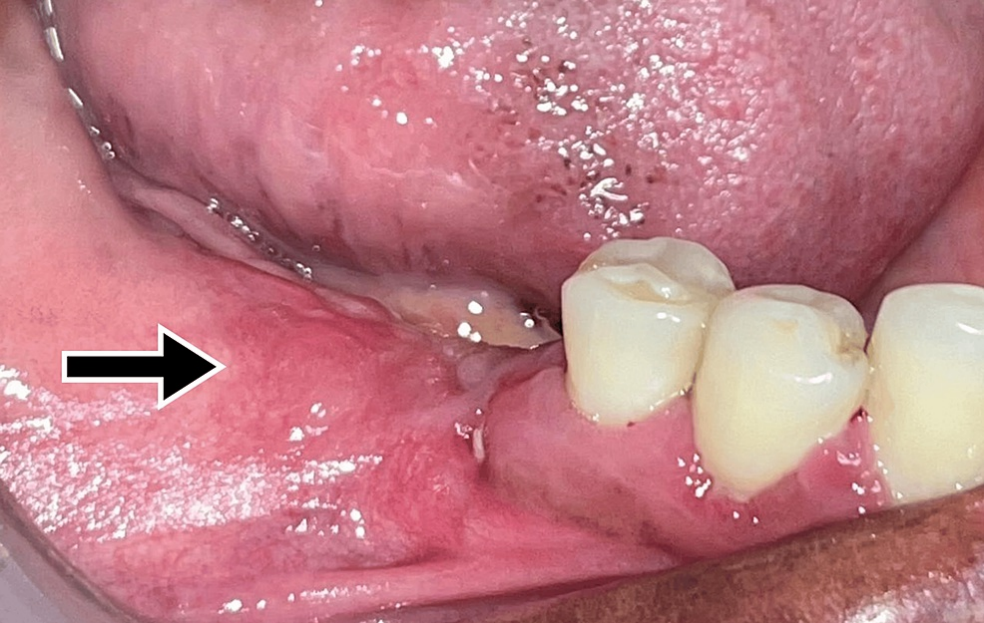
Four months post-operative follow-up

During the immediate post-operative period of seven days, uneventful healing was observed. The patient was kept on regular follow-up for the past four months and satisfactory healing was present (Figure 5). No recurrence was observed.

## Discussion

POF is a relatively common benign tumour of the oral cavity that primarily affects the gingiva or alveolar mucosa. Despite its non-neoplastic nature, POF can cause significant discomfort and affect oral health if left untreated [3]. There are two forms of POF, central and peripheral. The peripheral variant of ossifying fibroma emerges on the soft tissues enveloping the alveolar process, whereas the central form originates from the endosteum or the PDL adjacent to the root apex and grows from the medullary cavity of the bone [4]. POF typically presents as a firm, pink or red, nodular mass on the gingiva or alveolar mucosa. It may vary in size and can be either sessile or pedunculated. Some common symptoms associated with POF include swelling or enlargement of the gums, bleeding from the affected area, especially during brushing or eating, ulceration or erosion of the overlying mucosa, discomfort or pain, particularly when pressure is applied to the lesion and tooth mobility or displacement in severe cases [5].

The exact aetiology of POF remains unclear, although it is believed to arise from the PDL or gingival connective tissue in response to chronic irritation or trauma [6]. Factors such as poor oral hygiene, ill-fitting dental appliances, and hormonal changes may predispose individuals to the development of POF. Additionally, local irritants such as dental plaque, calculus and foreign objects lodged in the gingiva have been implicated in its pathogenesis [7].

The lesions' histopathology reveals stratified squamous epithelium overlying an extraordinarily dense mass of connective tissue, consisting of plump fibrocytes, fibrillar stroma and plump fibroblasts, as well as areas of mineralization and, occasionally, multinucleated giant cells nearby. Bone, cementum-like material or dystrophic calcifications can be found in the mineralization. Early ulcerated lesions typically exhibit dystrophic calcifications, but older, mature, non-ulcerated lesions display well-formed bone and cementum-like material [8].

The clinical presentation of POF can mimic other oral lesions, making differential diagnosis challenging. Conditions that may resemble POF include pyogenic granuloma, peripheral giant cell granuloma, fibroma and peripheral odontogenic fibroma. Pyogenic granuloma can occur anywhere in the oral cavity whereas POF occurs in the gingival and alveolar mucosa [9]. It is observed that peripheral giant cell granuloma occurs on the edentulous area; however, POF occurs on the edentulous area as observed in our case [10]. Histologically peripheral odontogenic fibroma contains giant cells whereas POF contains odontogenic epithelium and dysplastic dentin [11]. A thorough clinical examination, along with histopathological evaluation, is essential to differentiate POF from other oral pathologies accurately [12].

The management of POF usually involves surgical excision of the lesion followed by histopathological examination to confirm the diagnosis and ensure complete removal. Depending on the size and location of the tumour, various surgical techniques may be employed, including conventional scalpel excision, laser ablation or electrosurgery [13]. Neodymium-doped yttrium aluminum garnet (Nd:YAG) has been reported for the successful excision of POF with minimal bleeding. A case report has demonstrated the removal of a POF measuring 1 x 1.5 cm located on the maxillary right central incisor using a laser with no spontaneous bleeding and showed a minimal recurrence rate of up to one year [14]. The usage of electrocautery will help to achieve a bloodless field and making it useful for the excision of bigger POF lesions [15]. It is essential to remove the entire lesion along with a margin of healthy tissue to prevent recurrence. Adjunctive therapies such as topical or systemic medications may be prescribed to manage symptoms or prevent recurrence. Recurrence of POF can be due to the presence of local irritants, repeated injury to the same area and inadequate removal [16].

## Conclusions

Surgical excision is the curative management for POF. Plaque or irritation from the dental prosthesis should also be treated for the management of ossifying fibroma. If surgical excision is not performed, the lesion continues to grow and cause destruction of bone. Following surgical excision, the recall visit should be scheduled after one week. The patient should be on a regular follow-up for at least three months.
